# Classroom Active Breaks Effects on Attention and Concentration Performance Among Adolescents Attending Upper Secondary Schools in Southern Italy

**DOI:** 10.3390/sports14090404

**Published:** 2026-09-14

**Authors:** Domenico Martone, Daniela Vitucci, Andrea Gillo, Sara Dei, Loretta Francesca Cosco, Giuliana Valerio, Rosa Ghirelli, Andreina Alfieri, Annamaria Mancini, Stefania Orrù, Pasqualina Buono

**Affiliations:** 1 Department of Economics, Law, Cybersecurity and Sport Sciences, University Parthenope, 80133 Naples, Italy; daniela.vitucci@uniparthenope.it; 2 Department of Medical, Human Movement and Wellbeing Sciences, University Parthenope, 80133 Naples, Italy; andrea.gillo001@studenti.uniparthenope.it (A.G.); sara.dei@studenti.uniparthenope.it (S.D.); loretta.cosco@gmail.com (L.F.C.); giuliana.valerio@uniparthenope.it (G.V.); ghirelli@ceinge.unina.it (R.G.); andreina.alfieri@uniparthenope.it (A.A.); annamaria.mancini@uniparthenope.it (A.M.); stefania.orru@uniparthenope.it (S.O.); pasqualina.buono@uniparthenope.it (P.B.)

**Keywords:** active breaks, cognitive performance, sedentary behavior, school-based intervention, upper secondary school

## Abstract

Background: Physical inactivity and prolonged sedentary behavior have been associated with impaired cognitive function during adolescence. Although classroom-based active breaks (ABs) have shown promising effects in primary school children, evidence among adolescents attending upper secondary schools remains limited. This study evaluated the effects of a four-month classroom-based ABs program on attention and concentration performance and explored whether selected participant and lifestyle-related characteristics moderated the intervention response. Methods: In this cluster-randomized controlled trial, 529 adolescents (302 females, 227 males; 14.3 ± 0.9 years) from 39 classes attending four upper secondary schools in Southern Italy were included. Classes were randomly allocated to the intervention group (IG; 20 classes, *n* = 288) or control group (CG; 19 classes, *n* = 241). The IG participated in two daily 10 min classroom-based ABs for four months, whereas the CG followed the usual school routine. At baseline, anthropometric characteristics, sport participation, sleep duration, daily smartphone use, adherence to the Mediterranean Diet, and concentration performance (CP score), assessed by the d2-R Test of Attention, were evaluated; only CP was reassessed after the intervention. Results: The primary mixed-effects model showed significantly higher adjusted post-intervention CP in the IG than in the CG (adjusted between-group difference = 56.7 points, 95% CI 21.7–91.6; *p* = 0.003). The intervention effect remained significant in a fully adjusted model including sex, organized sport participation, sleep duration, smartphone use, and Mediterranean Diet adherence (*p* = 0.004). Exploratory moderator analyses provided no evidence that the intervention effect differed according to any of these characteristics after correction for multiple testing. Conclusions: A four-month classroom-based ABs program can improve concentration performance among adolescents. High intervention fidelity and student attendance support the feasibility of implementing structured ABs within the school setting over the intervention period. Further research is needed to determine the long-term effects and implementation of ABs program in secondary schools.

## 1. Introduction

Physical inactivity and sedentary behavior among children and adolescents represent major public health concerns worldwide. According to the World Health Organization (WHO), young people aged 5–17 years should accumulate at least 60 min of moderate-to-vigorous physical activity (MVPA) daily and limit prolonged sedentary behaviors whenever possible [1,2]. Nevertheless, global surveillance data indicate that more than 80% of adolescents fail to meet current physical activity (PA) recommendations, with the lowest participation rates observed among adolescents [3]. This developmental stage is characterized by a progressive decline in PA levels, increased sedentary time and a higher likelihood of sports dropout, particularly during the transition from lower to upper secondary education level [4,5].

School environments play a crucial role in shaping adolescents’ movement behaviors because students spend a substantial proportion of their waking hours in educational settings [6]. However, traditional school routines often involve prolonged periods of uninterrupted sitting, particularly during classroom lessons, contributing to sedentary behavior time accumulation throughout the school day [7].

Emerging evidence suggests that excessive sedentary time negatively affects not only physical health but also cognitive function, including attention, concentration, and academic engagement [8]. Given the growing concern regarding sedentary lifestyles among adolescents, schools have implemented interventions aimed at promoting movement and reducing sitting time without compromising academic instruction [8,9]. Among the multiple school-based PA strategies, Active Breaks (ABs) have received considerable scientific attention in recent years. ABs are short bouts of structured PA, typically lasting between 5 and 15 min, integrated into the school day during or between academic lessons to interrupt prolonged sitting and increase movement opportunities [10,11,12]. These activities may include aerobic and balance exercises, coordinative tasks, mobility drills, and muscle-strengthening movements that can be performed within the classroom environment with minimal equipment requirements [10,11]. The practical feasibility, low implementation costs, and compatibility with curricular demands make ABs particularly attractive for secondary school settings [11].

Beyond their contribution to increasing daily PA levels, ABs have been proposed as a promising strategy to enhance cognitive function and learning-related outcomes. Evidence suggests that both acute and chronic PA may support brain health and cognitive performance through several physiological pathways, including changes in cerebral blood flow, arousal, and neuroplasticity [13,14,15]. Consequently, school-based PA interventions have been investigated not only as health-promotion tools but also as potential strategies to support academic achievement and classroom behavior. Recent systematic reviews and meta-analyses have reported beneficial effects of ABs on several cognitive outcomes, including attention, on-task behavior, executive functions, and classroom engagement [12,16,17]. However, the current evidence remains heterogeneous regarding intervention characteristics, outcome measures, and participant age groups.

Recent reviews have increasingly examined the effects of ABs in adolescents and secondary school students [17,18]; nevertheless, the body of research involving adolescents in secondary schools remains limited compared to the abundance of literature on primary school children [11]. This distinction is relevant because adolescents may respond differently to PA interventions due to developmental changes in cognitive maturation, motivational regulation, academic demands, and health-related behaviors that characterize this developmental stage [19]. Therefore, findings derived from younger populations cannot be automatically generalized to secondary school students.

Several lifestyle-related factors may further influence cognitive functioning during adolescence and potentially interact with the effects of school-based PA interventions. Sleep duration is recognized as a major determinant of attention, executive functioning, and academic performance, whereas insufficient sleep has consistently been associated with poorer cognitive outcomes and behavioral difficulties [20,21]. Likewise, participation in organized sports and higher levels of physical fitness have been linked to favorable cognitive, psychosocial, and academic outcomes among adolescents [22,23]. Conversely, higher smartphone use has been associated with reduced attentional performance, impaired academic functioning, and adverse mental health outcomes [24,25]. Because these behaviors are modifiable and highly prevalent during adolescence, understanding their contribution may help explain individual variability in responsiveness to AB interventions.

Despite the growing international interest in ABs, evidence from Southern European adolescent populations remains limited. Furthermore, few studies have simultaneously examined the effectiveness of long-term classroom-based ABs program and the contribution of lifestyle-related behaviors to cognitive outcomes in secondary school students. To the best of our knowledge, no previous investigation has specifically explored these relationships among adolescents attending secondary schools in the Campania region of Southern Italy. Addressing this gap may contribute to a better understanding of how school-based AB interventions can be integrated into educational practice.

Therefore, the primary aim of the present study was to evaluate the effects of a four-month classroom-based ABs intervention on attention and concentration performance in adolescents attending upper secondary schools in the Campania region of Southern Italy. We hypothesized that students participating in the ABs program would demonstrate greater improvements in attention and concentration than those in the control group. As a secondary objective, we explored whether sex, organized sport participation, sleep duration, smartphone use, and adherence to the Mediterranean Diet moderated the intervention response.

## 2. Materials and Methods

### 2.1. Study Design

This cluster-randomized controlled trial involved students from four upper secondary schools located in different provinces of the Campania region (Italy). The activities were carried out at school, during the curricular hours of Physical Education (PE), by expert kinesiologists in collaboration with PE teachers. Participating classes (clusters) were randomly allocated to either an Intervention group (IG) or a Control group (CG). Students in the classes assigned to the IG participated in a four-month ABs program integrated into the regular school schedule, whereas the students in the CG followed their usual school routine. The intervention consisted of two daily ABs bouts, performed every school day during the second and fifth lesson periods. Each AB bout lasted 10 min and consisted of mobilization, stretching, strengthening, coordination and balance exercises performed by following music-based videos displayed on the interactive whiteboard under teacher supervision. Teachers were instructed to facilitate the immediate transition back to regular classroom activities following each AB bout and to minimize unnecessary social interaction during the resumption of the lesson.

The classroom ABs program was based on 12 freely available 10 min exercise videos selected from the official STRONG Nation^®^ YouTube channel (https://www.youtube.com/@WeAreSTRONGNation accessed on 29 November 2024). Videos were selected by the research team according to participants’ age, classroom feasibility, safety, involvement of large muscle groups, and suitability for performing bodyweight exercises within the classroom environment. The same set of videos was used throughout the four-month intervention and delivered to all intervention classes following the same sequential rotation (Videos 1–12), after which the sequence was restarted. Students followed the music-synchronized exercise rhythm provided by each video, with no individualized intensity prescription. To characterize the exercise intensity achieved during the ABs, a subsample of 150 students was randomly selected from the IG, with participants distributed as evenly as possible across intervention classes and schools. Approximately halfway through the intervention period, each selected student underwent one heart-rate assessment using a Polar H10 monitor (Polar Electro Oy, Kempele, Finland). Heart rate was continuously recorded throughout the entire 10 min ABs bout, and the mean HR over the session was calculated for each participant.

Teachers were instructed to start the scheduled video using the interactive whiteboard, supervise students throughout each AB bout, and record whether the scheduled session was completed. Student attendance was recorded individually at each AB using standardized attendance logs completed by the supervising teachers.

Before the intervention period, anthropometric characteristics and lifestyle-related behaviors were assessed at baseline. Attention and concentration performance (CP) score was evaluated at both baseline and post-intervention in all participants. All procedures were fully explained to all participants beforehand, ensuring a complete understanding of the study protocol. The study was reported in accordance with the applicable recommendations of the CONSORT extension for cluster-randomized trials.

### 2.2. Participants

A total of 529 adolescents (302 females and 227 males; aged 14.3 ± 0.9 years) participated in the study. Within each school, participating classes were first identified by the school and subsequently randomly allocated to either the IG or CG using a computer-generated random sequence. Twenty classes were allocated to the IG and 19 to the CG. The four schools contributed 12, 10, 9, and 8 classes, respectively. Randomization was not formally stratified by school, grade, or other participant characteristics and was performed by an investigator who was not involved in participant assessment or intervention supervision. All classes remained in their originally assigned condition throughout the four-month intervention, and no class withdrew after randomization.

No formal a priori sample-size calculation accounting for the clustered design was performed, as the sample size was determined by the number of eligible students available within the participating classes.

Students were recruited through participating schools and were eligible for inclusion if they regularly attended school activities and were able to participate in physical education and classroom-based PA sessions. Students with medical conditions or physical limitations preventing participation in PA were excluded from the study.

Parents or legal guardians were informed about the aims, procedures, potential benefits, and risks of the study and provided written informed consent prior to enrolment. In addition, written informed assent was obtained from all adolescent participants before the intervention. Each student was assigned an identification number to ensure anonymity during data collection and analysis. The study was conducted in accordance with the Declaration of Helsinki and was approved by the regional ethics committee of the University of Campania “Luigi Vanvitelli” (n. 0016488/i, approved on 1 June 2023).

### 2.3. Anthropometric Measures

Body height was measured to the nearest 0.1 cm using a portable stadiometer (SECA 213, Birmingham, UK), while body mass was measured to the nearest 0.1 kg using calibrated equipment (SECA 813, Birmingham, UK). Body Mass Index (BMI) was calculated as body mass divided by height squared (kg/m^2^). Participants’ overweight or obese status was classified according to the WHO age- and sex-specific cut-off points [26].

### 2.4. Questionnaire

Participants completed an anonymous self-administered questionnaire at baseline to collect information regarding selected lifestyle-related behaviors potentially associated with attention and concentration performance. Sleep duration was assessed by asking participants how many hours they usually slept per night and was categorized as <5 h/night, 6–8 h/night, or >8 h/night. Daily smartphone use was assessed by asking participants how many hours per day they usually used their smartphone and was categorized as <3 h/day (low), 3–5 h/day (medium), or >5 h/day (high). Organized sport participation was defined as regular participation outside school hours in activities provided by a sports club or association (Yes/No).

The KIDMED questionnaire consists of 16 items evaluating dietary habits consistent with the Mediterranean dietary pattern. Based on the total score obtained, participants were classified as having low, medium, or high adherence to the Mediterranean Diet according to established cut-off values [27].

Questionnaires were administered during regular school hours under the supervision of the research team (see Supplementary File S1).

### 2.5. Attention and Concentration Performance

Attention and concentration were assessed using the Italian version of the d2-R Test of Concentrated Attention, a standardized instrument designed to evaluate selective attention and the ability to process relevant information under time constraints [28].

The test requires participants to visually scan a series of symbols and identify predefined target stimuli while disregarding highly similar non-target stimuli. Specifically, students were asked to recognize the letter “d” marked with exactly two dashes and to ignore all other combinations. The task was organized into 14 consecutive rows of characters and was performed under timed conditions, with 20 s available for each row according to the standardized administration protocol.

Prior to testing, participants received verbal instructions and completed a short familiarization trial to ensure comprehension of the task requirements. All assessments were conducted collectively during school hours in a quiet classroom environment and under the supervision of trained researchers.

Among the indices provided by the d2-R, Concentration Performance (CP) was selected as the primary outcome measure. CP reflects the ability to maintain attentional focus while accurately discriminating target stimuli from distractors and controlling impulsive responses [28]. The CP score was calculated according to the procedures described in the test manual and represents the balance between speed and accuracy of performance. Specifically, the raw CP score is derived from the number of correctly identified target stimuli adjusted for commission errors and omissions, thereby reflecting the balance between speed and accuracy. Higher scores indicate better attentional efficiency and concentration capacity. Raw CP scores were used in all statistical analyses without transformation.

The d2-R has demonstrated satisfactory psychometric properties and is widely used in educational and research settings for the assessment of attentional functioning in adolescents and young adults [28]. The test was administered before the beginning of the intervention and repeated after the four-month intervention period under comparable testing conditions. Baseline and post-intervention assessments were administered during the same school lesson period. No ABs bout was performed on the assessment days, thereby avoiding the potential influence of an acute exercise bout on concentration performance. The d2-R tests were scored using participants’ identification codes without reference to group allocation.

### 2.6. Statistical Analysis

Continuous variables are presented as mean ± standard deviation (SD), whereas categorical variables are expressed as frequencies and percentages. Baseline differences between the IG and CG were assessed using independent-samples t-tests for continuous variables and Pearson’s chi-square tests for categorical variables. When the assumptions of normality were not met, the Mann–Whitney U test was used. Baseline between-group differences were additionally quantified using absolute standardized mean differences (|SMD|). For continuous variables, |SMD| was calculated as the absolute value of Cohen’s d; for categorical variables, |SMD| was calculated using standardized differences between proportions, separately for each category when more than two levels were present.

To evaluate the intervention effect while accounting for the cluster-randomized design, a linear mixed-effects model was fitted with the post-intervention CP score as the dependent variable, group and school as fixed effects, baseline CP score and age as covariates, and class as a random intercept. A secondary fully adjusted model additionally included sex, organized sport participation, sleep duration, smartphone use, and Mediterranean Diet adherence. Exploratory moderator analyses were conducted by separately testing Group × Sex, Group × Sport Participation, Group × Sleep Duration, Group × Smartphone Use, and Group × Mediterranean Diet Adherence interactions. The corresponding omnibus *p*-values were adjusted for multiple testing using the Holm procedure.

Models were estimated using restricted maximum likelihood (REML) with Satterthwaite-adjusted degrees of freedom. Model assumptions were assessed by inspection of residual Q–Q and residual-versus-fitted plots and by examination of the distribution of class-level random effects. The intraclass correlation coefficient (ICC) was calculated to quantify clustering at the class level. Adjusted between-group differences and estimated marginal means were reported with 95% confidence intervals.

Jamovi software (version 2.7.34) was used for the analyses [29]. Statistical significance was set at *p* < 0.05.

## 3. Results

### 3.1. Participant Flow and Baseline Characteristics

**Figure 1:**
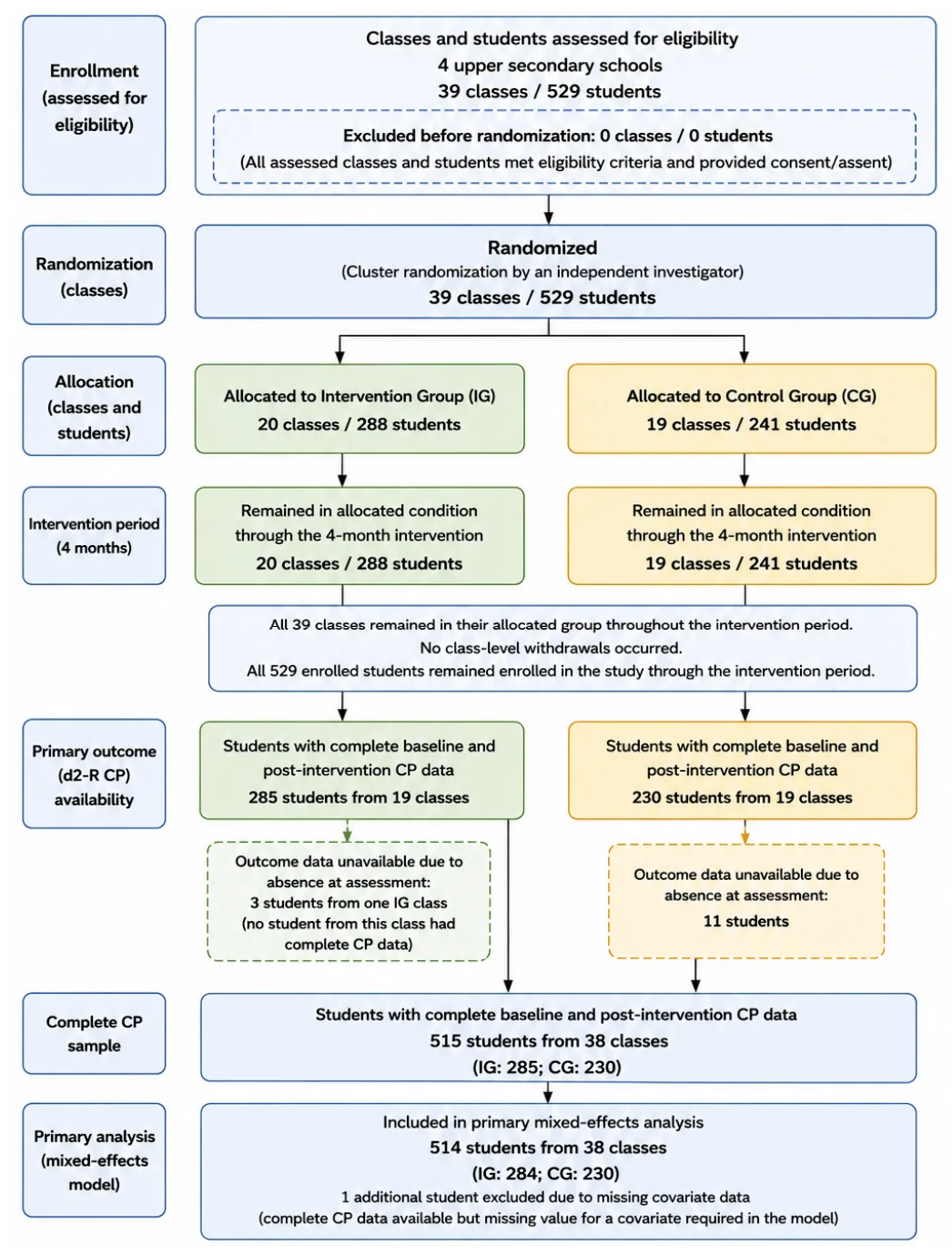
Participant and class flow diagram (cluster-randomized controlled trial).

All 39 randomized classes and all 529 enrolled students remained in the study throughout the four-month intervention period, with no class-level withdrawals. Complete baseline and post-intervention CP data were available for 515 students (285 IG and 230 CG); 14 students (3 IG and 11 CG) were absent during one of the d2-R assessment sessions. The primary mixed-effects analysis included 514 students from 38 classes because one additional participant had missing covariate data (Figure 1).

Baseline characteristics of the 529 participants (IG, *n* = 288; CG, *n* = 241) are presented in Table 1.

Significant between-group differences were observed for age, body weight, CP score, and the proportion of students reporting more than 8 h of sleep per night. Students in the CG had a significantly higher mean age (*p* < 0.001), higher body weight (*p* = 0.015), and higher baseline CP scores (*p* = 0.040) than the IG. In addition, a greater proportion of students in the IG reported sleeping more than 8 h per night compared to the CG (*p* = 0.014). The distribution of daily smartphone use categories also differed significantly between groups (*p* = 0.049). No significant between-group differences were found in sex distribution, height, BMI, prevalence of overweight/obesity, sport participation, overall Mediterranean Diet adherence, or in the other sleep categories (all *p* > 0.05).

### 3.2. Intervention Fidelity

Intervention fidelity was high throughout the study. Of the 192 classroom ABs bout scheduled during the four-month intervention period, 180 (93.8%) were successfully delivered. Mean student attendance across the ABs bout was approximately 93.1%. Heart-rate monitoring conducted halfway through the intervention in the subsample of 150 IG students showed a mean 10 min session HR of 155 ± 5.3 bpm, a value compatible, at the group level, with moderate-to-vigorous exercise intensity in adolescents of comparable age [30].

### 3.3. Concentration Performance

Descriptive baseline, post-intervention, and change CP scores for the IG and CG are presented in Table 2.

### 3.4. Effects of the Active Breaks Intervention on Concentration Performance

The primary linear mixed-effects model, accounting for clustering at the class level and adjusting for baseline CP score, age, and school, showed a significant effect of group on post-intervention CP (F (1, 34.6) = 10.16, *p* = 0.003). The adjusted post-intervention CP score was higher in the IG (estimated marginal mean = 151.3, 95% CI 127.1–176.0) than in the CG (94.7, 95% CI 68.3–121.0), with an adjusted between-group difference of 56.7 points (95% CI 21.7–91.6). The class-level ICC was 0.275 (Table 3).

In the secondary fully adjusted model, which additionally included sex, organized sport participation, sleep duration, smartphone use, and Mediterranean Diet adherence, the group effect remained significant (F (1, 34.2) = 9.34, *p* = 0.004), with an adjusted between-group difference of 54.7 points (95% CI 19.5–89.8). None of the additional participant or lifestyle-related factors showed a significant main effect (all *p* > 0.05).

### 3.5. Exploratory Moderator Analyses

Exploratory moderator analyses provided no evidence that the intervention effect differed according to sex (Group × Sex: F (1, 488.5) = 1.42, *p* = 0.234), organized sport participation (Group × Sport Participation: F (1, 479.4) = 0.03, *p* = 0.870), sleep duration (Group × Sleep Duration: F (2, 470.9) = 1.21, *p* = 0.299), smartphone use (Group × Smartphone Use: F (2, 475.1) = 2.74, *p* = 0.066), or Mediterranean Diet adherence (Group × Mediterranean Diet Adherence: F (2, 470.9) = 0.37, *p* = 0.693). Following Holm correction for the five interaction tests, none of the moderator effects were statistically significant (all adjusted *p* ≥ 0.330). Consequently, no post hoc pairwise comparisons were performed.

## 4. Discussion

The present study evaluated the effects of a four-month classroom-based ABs program on concentration performance among adolescents attending upper secondary schools in the Campania Region, Italy.

We found that students participating in the ABs program achieved significantly higher post-intervention CP scores than their peers in the CG, even after adjustment for baseline concentration performance, age, school, and clustering at the class level. The adjusted between-group difference was 56.7 points (95% CI 21.7–91.6), and a comparable effect was observed in the secondary fully adjusted model including sex, organized sport participation, sleep duration, smartphone use, and Mediterranean Diet adherence. These findings support our primary hypothesis and reinforce the evidence that integrating ABs into the regular school day may represent an effective strategy for enhancing cognitive function during adolescence.

Our findings are consistent with some systematic reviews indicating that classroom-based ABs can positively influence attention, executive functions, and classroom behavior in school-aged children and adolescents, although the overall evidence remains heterogeneous because of differences in intervention characteristics, outcome measures, and educational settings [12,16,17,18]. Nevertheless, the available evidence remains heterogeneous, partly because of differences in intervention characteristics, duration, intensity, cognitive outcome measures, and educational settings [16,17,18]. Importantly, much of the available evidence has focused on primary school children, whereas studies involving adolescents attending upper secondary schools remain comparatively limited [11,17,18]. This distinction is relevant because adolescence is characterized by declining physical activity, increasing sedentary behavior, changes in cognitive maturation, and increasing academic demands [3,4,19]. Accordingly, findings derived from younger children cannot necessarily be generalized to upper secondary school students. The present study extends the existing literature by showing that a four-month classroom-based ABs program implemented in adolescents attending upper secondary schools can improve concentration performance under real-world educational conditions.

Several physiological and neurocognitive mechanisms proposed in the literature may potentially contribute to the association between physical activity and cognitive performance. Short bouts of physical activity have been associated with changes in cerebral blood flow and cortical arousal [15,31]. Acute exercise has also been associated with transient changes in catecholaminergic neurotransmission, which may facilitate attentional and executive processes [15]. Furthermore, interrupting prolonged sedentary behavior with brief periods of physical activity may help maintain prefrontal cortical function and cognitive performance [32,33,34]. These mechanisms provide plausible biological explanations for the cognitive effects reported following PA interventions; however, none of these neurophysiological pathways were directly assessed in the present study, and they should therefore be regarded as potential rather than demonstrated mechanisms underlying the observed improvement in CP.

The exploratory analyses did not provide evidence that the effect of the ABs program on concentration performance differed according to sex, organized sport participation, sleep duration, smartphone use, or Mediterranean Diet adherence. This suggests that, within the characteristics and categories examined in the present sample, no clear subgroup-specific response to the intervention could be identified. However, the absence of statistically significant interactions should not be interpreted as evidence that these characteristics are unrelated to cognitive performance or responsiveness to PA. Several lifestyle and participant characteristics have previously been associated with cognitive functioning during adolescence and may remain relevant when considering the complex determinants of attentional performance [19,25]. In particular, organized sport participation has previously been associated with cognitive function, academic achievement, and psychosocial outcomes in adolescents [22,23]. In the present study, however, organized sport participation did not significantly modify the effect of the ABs program. Importantly, the questionnaire assessed regular participation in structured extracurricular sport rather than overall habitual PA. Adolescents classified as non-participants in organized sport may therefore still have engaged in substantial informal or recreational PA, while sport participation itself does not provide a comprehensive estimate of total activity or sedentary behavior. Future studies using objective measures such as accelerometry may better establish whether habitual PA or sedentary time influences cognitive responsiveness to classroom-based AB interventions.

Smartphone use also represents a potentially relevant lifestyle characteristic during adolescence. Recent evidence indicates that smartphone use during school hours is common and that more frequent smartphone checking may be associated with poorer cognitive control [35]. Problematic short-video use has also been associated with poorer attentional control in EEG-based research [36], while broader digital-media exposure has been linked to several aspects of executive functioning, including attention, working memory, inhibitory control, and cognitive flexibility [37]. In the present study, however, smartphone use did not significantly moderate the intervention effect. Moreover, smartphone exposure was assessed only through broad self-reported duration categories and did not distinguish between educational use, social media, gaming, passive viewing, or problematic use. Consequently, the present findings cannot determine whether specific patterns or purposes of smartphone use influence concentration performance or responsiveness to ABs.

The present study has several strengths that enhance the robustness and practical relevance of its findings. First, it contributes to the still limited evidence on the effects of classroom-based ABs in adolescents attending upper secondary schools, a population that has received considerably less attention than primary school children despite the marked decline in PA and increase in sedentary behavior observed during adolescence [3,4,11]. Second, the intervention was implemented over a relatively long period (four months) under real-world school conditions, with high intervention fidelity (93.8% of planned ABs bout delivered) and mean student attendance of approximately 93.1%. The high intervention fidelity and student attendance observed throughout the study support the feasibility of implementing classroom-based ABs within the school setting over the four-month intervention period. Third, the cluster-randomized design reflects the real-world organization of the school setting, in which students are naturally grouped within classes. The main intervention effect was also consistent after additional adjustment for selected participant and lifestyle-related characteristics. Finally, exercise intensity was descriptively characterized through heart-rate monitoring in a subsample of intervention participants.

Nevertheless, several limitations should be acknowledged. Potential contamination between IG and CG classes within the same schools could not be excluded. Moreover, randomization was performed at the class level without formal stratification by school or baseline participant characteristics, which may have contributed to the observed baseline imbalance between groups. Participants and teachers could not be blinded to group allocation because of the nature of the intervention, although d2-R tests were scored using participant identification codes without reference to group allocation. Teacher acceptability and receptivity toward the ABs program, as well as its potential impact on regular teaching activities and instructional time, were not formally assessed.

Heart-rate monitoring was performed only once in a subsample of the IG, and individual HRmax was not assessed; therefore, HR should be considered a descriptive group-level indicator of session intensity rather than a precise measure of individual relative exercise intensity. Lifestyle-related variables were self-reported, and sleep duration and smartphone use were assessed using broad categories. Habitual PA and sedentary time were not objectively assessed, and organized sport participation should not be interpreted as a measure of overall PA level or sedentary behavior. Repeated administration of the d2-R may also have introduced a practice effect, although the same testing procedure was applied to both groups. In addition, the moderator analyses were exploratory and involved multiple testing, limiting the strength of conclusions concerning subgroup-specific responses. Finally, although participants were recruited from different provinces of the Campania region, caution is warranted when generalizing the findings to other educational systems or cultural contexts. No longer-term follow-up assessment was performed; therefore, whether the observed improvement in concentration performance persists after completion of the intervention remains unknown.

Future research should investigate the long-term effects and implementation of classroom-based ABs program in more diverse educational settings. Studies incorporating objective measures of habitual physical activity and sedentary behavior, more detailed assessments of digital-media exposure, and formal evaluations of teacher acceptability, implementation burden, and instructional impact would further clarify the effectiveness and feasibility of ABs in upper secondary schools.

## 5. Conclusions

The present study indicates that a four-month classroom-based ABs program can improve concentration performance among adolescents attending upper secondary schools. The intervention effect remained significant after accounting for baseline concentration performance, age, school, and class-level clustering, and it was consistent after additional adjustment for selected participant and lifestyle-related characteristics.

Exploratory analyses provided no evidence that the intervention effect differed according to sex, organized sport participation, sleep duration, smartphone use, or Mediterranean Diet adherence. The high intervention fidelity and student attendance observed over the four-month period support the feasibility of implementing structured ABs within the school setting, although their long-term implementation and sustainability remain to be established.

Future research should investigate the long-term effects and implementation of ABs in more diverse educational settings and incorporate objective measures of habitual PA and sedentary behavior.

## Figures and Tables

**Table 1:** Baseline characteristics of participants.

Variable (Units)	Total (*n* = 529)	IG (*n* = 288)	CG (*n* = 241)	*p* Value	|SMD|
Age (years)	14.3 ± 0.9	14.0 ± 1.0	14.5 ± 0.7	<0.001	0.567
Sex (M/F, %)	42.9/57.1	41.6/58.4	44.4/55.6	0.523	0.055
Weight (kg)	62.8 ± 14.7	61.4 ± 13.8	64.6 ± 15.6	0.015	0.397
Height (cm)	163.5 ± 8.0	163.0 ± 8.3	164.3 ± 7.6	0.070	0.340
BMI (kg/m^2^)	23.3 ± 4.8	23.0 ± 4.5	23.7 ± 5.3	0.114	0.319
Overweight/obese	43%	22.2%	20.8%	0.149	0.003
Daily smartphone use					
<3 h/day (low)	11.8%	14.2%	9.6%	0.049	0.174
3–5 h/day (medium)	45.1%	44.4%	45.2%	0.857	0.017
>5 h/day (high)	43.1%	41.4%	45.2%	0.156	0.095
Sport participation	57.3%	56.6%	56.4%	0.954	0.004
Sleep duration					
<5 h	4.8%	3.5%	6.2%	0.139	0.129
6–8 h	64.3%	61.5%	68.5%	0.089	0.150
>8 h	30.9%	35.0%	25.3%	0.014	0.217
Mediterranean Diet adherence					
-Low	42.5%	44.4%	40.2%	0.331	0.085
-Medium	48%	47.6%	48.5%	0.823	0.020
-High	9.5%	8.0%	11.3%	0.208	0.109
CP score	62.3 ± 102.3	54.0 ± 99.0	72.6 ± 105.5	0.040	0.356

Note: Data are presented as mean ± standard deviation (SD) for continuous variables and percentages for categorical variables. *p* values refer to between-group comparisons (intervention group vs. control group). IG = Intervention Group; CG = Control Group; BMI = Body Mass Index; CP = Concentration Performance; SMD = Standardized Mean Difference. Absolute SMD values (|SMD|) are reported for all baseline characteristics.

**Table 2:** Concentration performance scores at baseline, post-intervention, and changes from baseline.

Group	Baseline CP	Post CP	Change
IG	54.0 ± 99.0	149.9 ± 96.2	95.9 ± 104.6
CG	72.6 ± 105.5	95.8 ± 99.4	23.2 ± 125.7

Data are presented as mean ± SD. Change was calculated as post-intervention minus baseline CP score. CP, Concentration Performance; IG, Intervention Group; CG, Control Group.

**Table 3:** Primary linear mixed-effects model for post-intervention concentration performance.

Fixed Effect	Estimate	SE	95% CI	F (df1, df2)	*p* Value
Group (IG vs. CG)	56.66	17.77	21.74 to 91.58	10.16 (1, 34.6)	0.003
Baseline CP score	0.323	0.039	0.246 to 0.400	67.84 (1, 506.8)	<0.001
Age	−3.03	4.47	−11.81 to 5.74	0.46 (1, 504.6)	0.497
School	—	—	—	0.74 (3, 33.1)	0.536

Note: CP, concentration performance; IG, Intervention Group; CG, Control Group; CI, confidence interval; SE, standard error. The model included post-intervention CP score as the dependent variable, group and school as fixed effects, baseline CP score and age as covariates, and class as a random intercept. For Group, the estimate is presented as IG − CG and represents the adjusted between-group difference in post-intervention CP. The class-level random-intercept variance was 2345 (SD = 48.4), the residual variance was 6174 (SD = 78.6), and the intraclass correlation coefficient (ICC) was 0.275. The analysis included 514 students from 38 classes.

## Data Availability

The data supporting the findings of this study are available from the corresponding author upon reasonable request.

## Supplementary Materials

The following supporting information can be downloaded at https://www.mdpi.com/article/10.3390/sports14090404/s1. Supplementary File S1: Questionnaire used to assess sleep duration, daily smartphone use, organized sport participation, and adherence to the Mediterranean Diet (KIDMED).
