# Supplementary material for: Classroom Active Breaks Effects on Attention and Concentration Performance Among Adolescents Attending Upper Secondary Schools in Southern Italy
Source: Sports (Basel). 2026 Sep 14;14(9):404. doi: 10.3390/sports14090404 (PMC13611686; doi:10.3390/sports14090404)
Supplement: Supplementary file 1 [file sports-14-00404-s001.zip › sports-4476772-supplementary.pdf]

FOR EXAMINERS' USE ONLY

---

1. Are you a boy or a girl?
Male Female

2. How old are you? _______________ years

3. What type of school and class do you attend (example: classical high school - second year)?

---

4. Generally, how many hours do you sleep each night?
less than 5 hours 6 to 8 hours more than 8 hours

5. How many hours per day do you usually use your smartphone?

less than 3 hours per day
between 3 and 5 hours per day
more than 5 hours per day

6. Outside school hours, do you regularly participate in sport through a sports club/association (e.g., football, basketball, volleyball, etc.)?
Yes No

ADHERENCE TO THE MEDITERRANEAN DIET (KIDMED)

1. Do you eat a piece of fruit or drink fruit juice every day?
Yes No

2. Do you eat a second piece of fruit every day?
Yes No

3. Do you eat raw or cooked vegetables regularly once a day?
Yes No

4. Do you eat raw or cooked vegetables regularly more than once a day?
Yes No

5. Do you eat fish regularly (at least 2-3 times a week)?
Yes No

6. Do you go to a fast-food restaurant (hamburgers) more than once a week?
Yes No

7. Do you eat legumes (peas, beans, lentils, chickpeas, etc.) more than once a week?
Yes No

8. Do you eat pasta or rice almost every day (5 or more times a week)?
Yes No

9. For breakfast, do you eat cereals, bread, or rusks?
Yes No

10. Do you eat nuts (walnuts, hazelnuts, almonds, etc.) regularly (at least 2-3 times a week)?
Yes No

11. Do you use olive oil at home for seasoning?
Yes No

12. Do you usually have breakfast?
Yes No

13. For breakfast, do you consume milk or dairy products (yogurt, milk, etc.)?
Yes No

14. For breakfast, do you eat packaged pastries/snack cakes and biscuits?
Yes No

15. Do you consume 2 yogurts and/or portions of cheese daily?
Yes No

16. Do you eat sweets and candies several times a day?
Yes No

THANK YOU FOR YOUR COOPERATION!
